# Supplementary material for: A second DNA binding site in human BRCA2 promotes homologous recombination
Source: Nat Commun. 2016 Sep 15;7:12813. doi: 10.1038/ncomms12813 (PMC5027613; doi:10.1038/ncomms12813)
Supplement: Supplementary Information — Supplementary Figures 1-10, Supplementary Table 1, Supplementary Methods and Supplementary References [file ncomms12813-s1.pdf]

Supplementary Information

Supplementary Figures and Legends

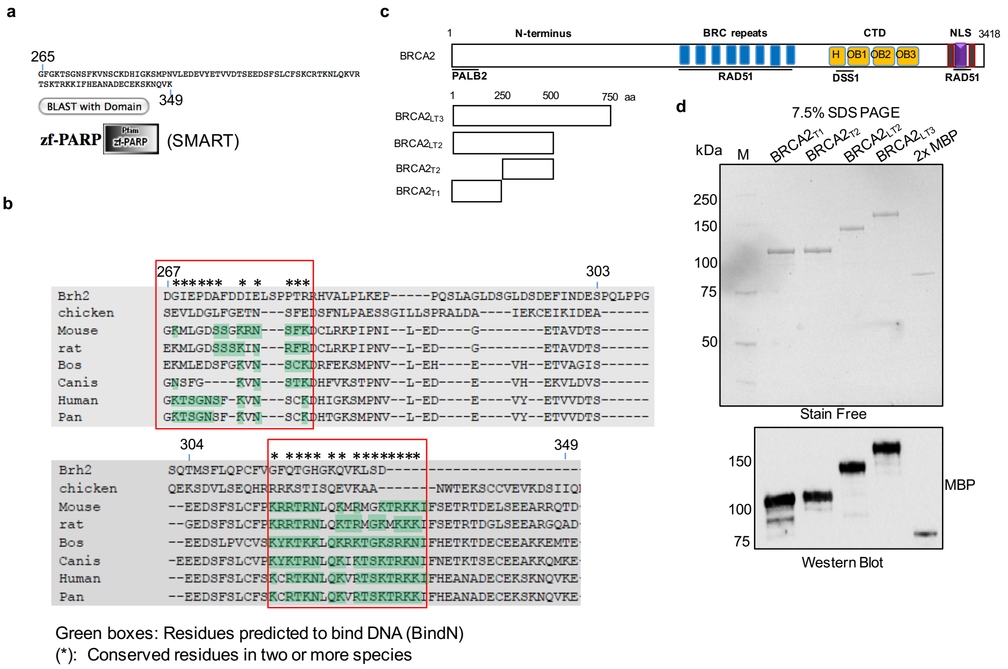

Supplementary Figure 1: Bioinformatics analysis of the N-terminal DNA binding region in BRCA2 and purified fragments. a, SMART result showing the putative zf-PARP like domain in BRCA2**. b,** Multiple alignment using the query sequence indicated in **a** showing the probable DNA binding residues in the indicated species as defined by BindN online software (in green). The numbers corresponds to the human protein sequence. **c**, Schematic representation of BRCA2 and the fragments derived from the N-terminal region used in this work. **d**, Purified fragments of the N-terminal region of BRCA2 shown in a SDS-PAGE gel and detected by stain-free imaging (Bio-Rad) and by western blot revealed with an antibody against the MBP tag.

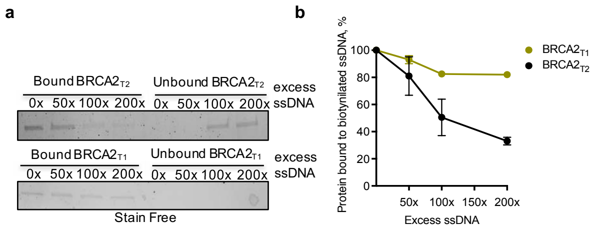

Supplementary Figure 2: BRCA2$_{T2}$ but not BRCA2$_{T1}$ binds DNA. a, SDS-PAGE showing a pull down experiment with BRCA2$_{T2}$ or BRCA2$_{T1}$ using biotinylated ssDNA (dT$_{80}$) immobilized on streptavidin magnetic beads challenged with the indicated excess concentration of non-biotinylated ssDNA (dT$_{40}$). **b**, quantification of a. Error bars, s.d. (n = 3).

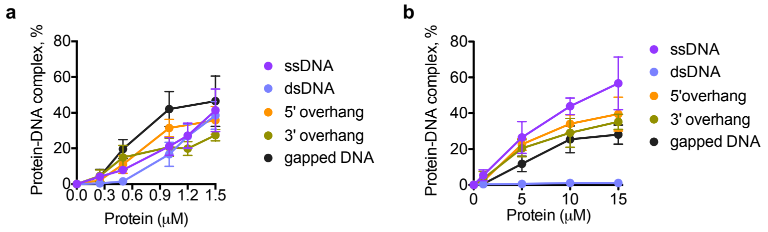

Supplementary Figure 3: BRCA2$_{T2}$ binds dsDNA and shows similar preference for dsDNA containing structures and ssDNA as opposed to the CTD. a, Comparison of the affinities of BRCA$_{T2}$ or **b**, CTD for binding to the different DNA substrates utilized in Figure 2. Error bars, s.d. (n = 3).

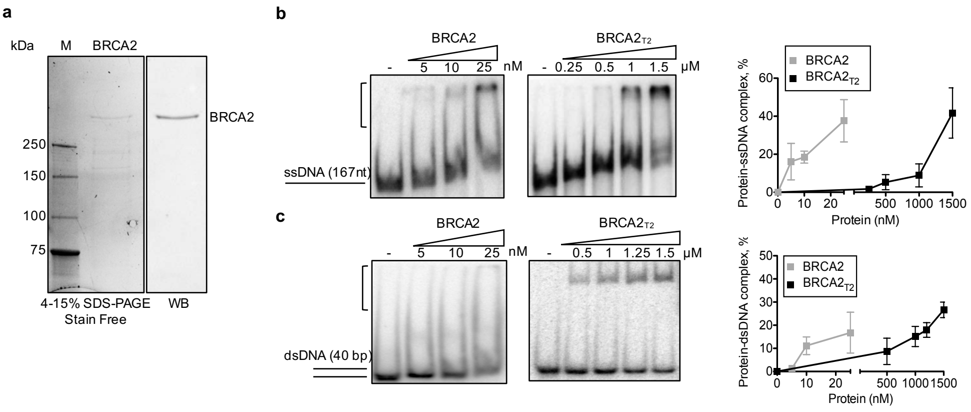

Supplementary Figure 4: BRCA2$_{T2}$ and BRCA2 binding to ssDNA and dsDNA. a. BRCA2 tagged with GFP-MBP at the N-terminus was purified from human cells and analyzed by SDS-PAGE. BRCA2 (0.6 µg) was loaded on a precast 4-15% SDS-PAGE gel and detected by stain free imaging. Western blot of the same purified BRCA2 protein using an antibody specific for the central region of BRCA2 (OP95, EMD). M, size standards. **b**. Autoradiogram and quantification of EMSA of BRCA2$_{T2}$ or BRCA2 binding to ssDNA (167 nt). **c**. Autoradiogram and quantification of EMSA of BRCA2$_{T2}$ or BRCA2 binding to dsDNA (40bp). Error bars, s.d. (n=3).

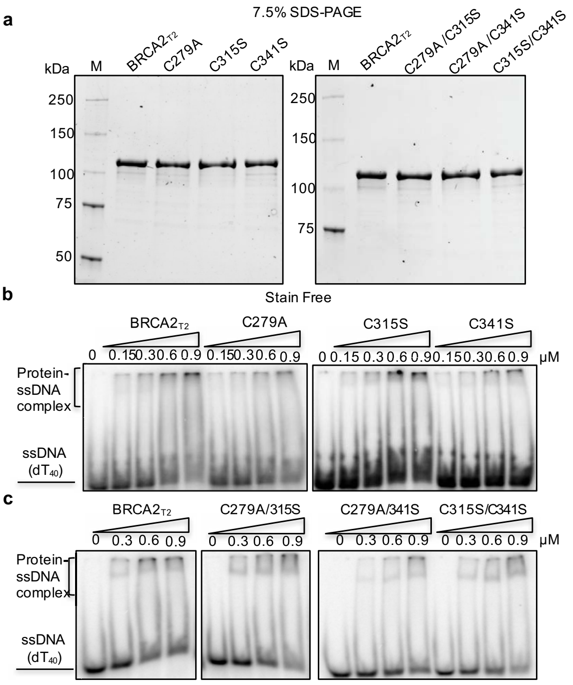

Supplementary Figure 5: ssDNA binding activity of single or double cysteine mutated BRCA2$_{T2}$. a, 1 μg of each purified wild type or mutated BRCA2$_{T2}$ fragment as indicated is shown in an SDS-PAGE gel and detected by stain-free imaging. **b**, Autoradiogram of EMSA of BRCA2$_{T2}$ or the single mutants indicated binding to ssDNA and quantified in Figure 3b. **c**, Autoradiogram of EMSA of BRCA2$_{T2}$ or the indicated double mutants and quantified in Figure 3c.

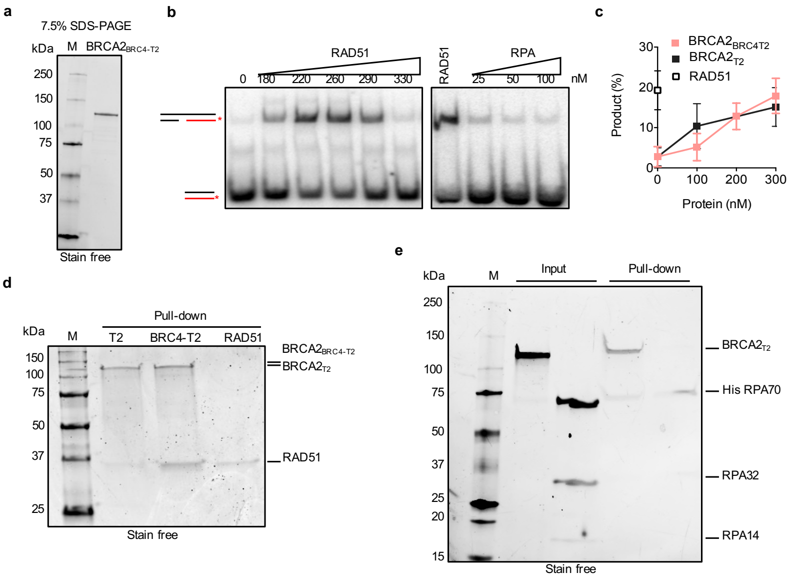

Supplementary Figure 6: BRCA2$_{T2}$ does not bind RAD51 nor RPA. a, SDS PAGE gel showing purified BRCA2$_{BRC4\text{-}T2}$. **b**, Autoradiogram of DNA strand exchange reaction using a 3’ ssDNA overhang substrate at the indicated increasing concentrations of RAD51 (left) or RPA (right). **c**, Comparison of the levels of DNA strand exchange reaction stimulation by BRCA2$_{BRC4\text{-}T2}$ (Fig. 4a, b) and BRCA2$_{T2}$ (Fig. 4c). Error bars, s.d. (n=3). **d**, Pull-down of purified BRCA2$_{BRC4\text{-}T2}$ or BRCA2$_{T2}$ and RAD51. **e**, Pull down of purified BRCA2$_{T2}$ and RPA.

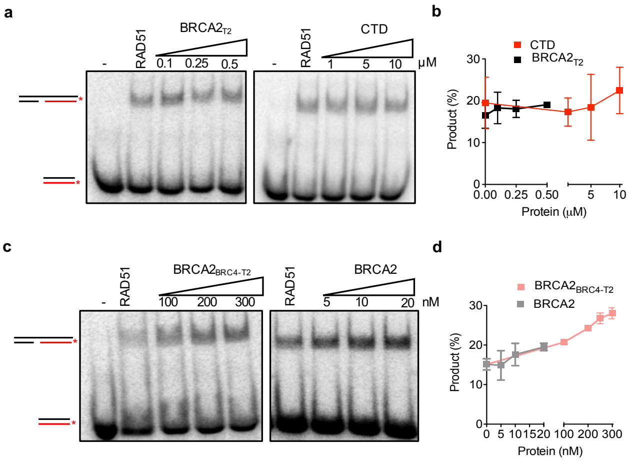

Supplementary Figure 7: BRCA2$_{BRC4-T2}$ but not BRCA2$_{T2}$ nor CTD can stimulate the DNA strand exchange activity of RAD51 in the absence of RPA. a, Autoradiogram of a DNA strand exchange reaction in the presence of RAD51 alone or with increasing concentrations of BRCA2$_{T2}$ or CTD. **b**, Quantification of **a**. **c**, Autoradiogram of a DNA strand exchange reaction in the presence of RAD51 alone or with increasing concentrations of BRCA2$_{BRC4-T2}$ or BRCA2. **d**, Quantification of **c**. Error bars, s.d. (n = 3).

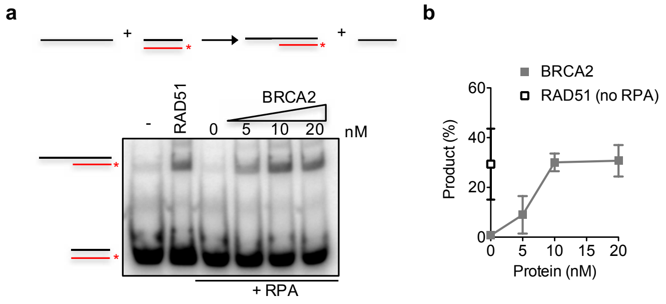

Supplementary Figure 8: BRCA2 stimulates the DNA strand exchange activity of RAD51 using a ssDNA substrate

a, Autoradiogram of a DNA strand exchange reaction in the presence of RAD51 alone,

RPA and RAD51, or RPA, RAD51 and increasing concentrations of BRCA2 using a ssDNA substrate. **b**, Quantification of **a**. Error bars, s.d. (n = 3).

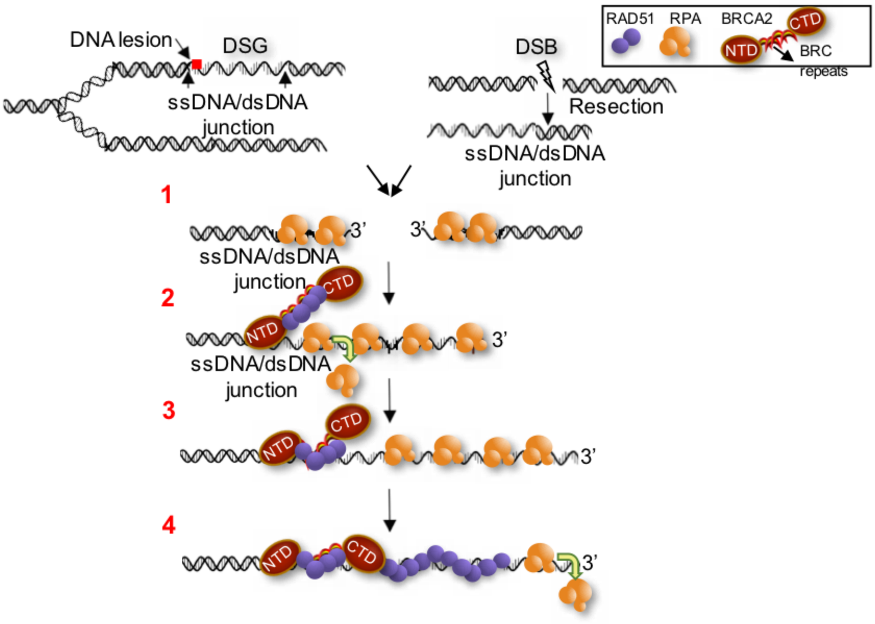

Supplementary Figure 9. Model for BRCA2 NTD and CTD coordination in the context of homologous recombination. (1) RPA binds the ssDNA generated upon resection of a DSB or the ssDNA flanking a DNA lesion (red square) during replication. (2) NTD binds at the dsDNA/ssDNA junction, facilitating the loading of RAD51 onto RPA-coated ssDNA by class I of the BRC repeats (BRC1-4)[1]. (3) Class II BRC repeats (5-8) stabilize the nascent RAD51 nucleoprotein filament [1]. (4) CTD binds along the ssDNA and actively facilitates the displacement of RPA[2] allowing multiple nucleation events and filament extension of RAD51. These coordinated activities allow subsequent homology pairing and DNA strand exchange required for the template-driven repair in the context of both a DSB or in daughter strand gap repair in the context of replication.

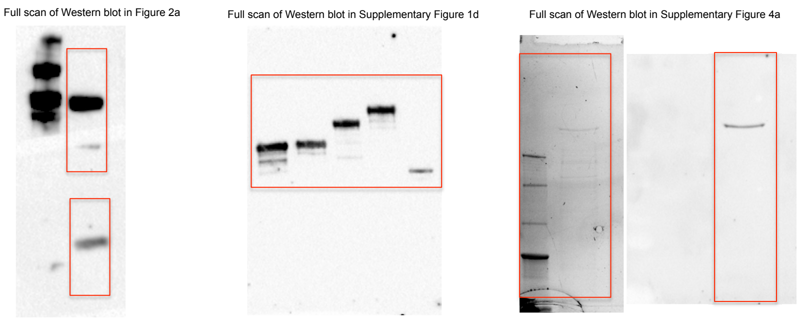

Supplementary Figure 10. Full scans of western blots in the main text and in supplementary section.

Supplementary Table

**Primer**	**Purpose**	**Sequence (5'-3')**
oAC403	For EMSA 42-mer 5' to anneal to 167-mer (oAC423)	CGGATATTTCTGATGAGTCGAAAAATTATCTTGATAAAGC AG
oAC379	dT_{40} for EMSA and biotin pull-down	TTTTTTTTTTTTTTTTTTTTTTTTTTTTTTTTTTTTTTTT
Biotin-modified dT_{80}	For biotin pull-down	TTTTTTTTTTTTTTTTTTTTTTTTTTTTTTTTTTTTTTTT TTTTTTTTTTTTTTTTTTTTTTTTTTTTTTTTTTTTTTTT
oAC405	40mer for EMSA, anneals with oAC406	TAATACAAAATAAGTAAATGAATAAACAGAGAAAATAAA G
oAC406	40mer for EMSA, anneals with oAC405	CTTTATTTTCTCTGTTTATTCATTTACTTATTTTGTATTA
oAC423	167mer for EMSA	CTGCTTTATCAAGATAATTTTTCGACTCATCAGAAATATCC GTTTCCTATATTTATTCCTATTATGTTTTATTCATTTACTTAT TCTTTATGTTCATTTTTTATATCCTTTACTTTATTTTCTCTGT TTATTCATTTACTTATTTTGTATTATCCTTATCTTATTTA
oAC490	42mer for 3't substrate with 167mer EMSA (oAC423)	TAAATAAGATAAGGATAATACAAAATAAGTAAATGAATA AAC
oAC491	SDM C279A in $BRCA2_{T2}$	AAG TAA ATA GCG CCA AAG ACC ACA TTG GAA AGT CAA TGC CAA ATG

oAC492	SDM C279A in $BRCA2_{T2}$	AAG TAA ATA GCG CCA AAG ACC ACA TTG GAA AGT CAA TGC CAA ATG
oAC493	SDM C341S in $BRCA2_{T2}$	CTTGGTTTTTAGATTTTTCACTTTCATCAGCGTTTGCTTCATGG
oAC494	SDM C341S in $BRCA2_{T2}$	CCATGAAGCAAACGCTGATGAAAGTGAAAAATCTAAAAACCAAG
oAC520	SDM C315S in $BRCA2_{T2}$	GTTTTTCATTATGTTTTTCTAAAAGTAGAACAAAAAATCTACAAAAAG
oAC521	SDM C315S in $BRCA2_{T2}$	CTTTTTGTAGATTTTTTGTTCTACTTTTAGAAAAACATAATGAAAAAC
oAC596	PCR for Gibson assembly on pAC138 to make NotI 2NLS BRC4	TTCCAGGGGCCCGGCGGCCGCGATCCAAAAAAGAAGAGAAAGG
oAC597	PCR for Gibson assembly on pAC138 to make NotI 2NLS BRC4	GTGTTTTCACTTTGCTCTTTTTCATCAAAAAGG
oAC598	PCR Gibson assembly on pAC098 for overlap with BRC4	AAAGAGCAAAGTGAAAACACAAATCAAAGAG
oAC599	PCR Gibson assembly on pAC098 for $BRCA2_{T2}$ stop	GCTGATTATGATCTAGACTCGAGTTAACCCTGAAATGAAGAAGC

Supplementary Table 1. List of primers and oligonucleotides used in this study.

Supplementary Methods

Secondary structure prediction and alignment

Using SMART secondary structure prediction tool (http://smart.embl.de/) we identified a putative zinc finger-PARP-like domain between amino acids 265-349 of BRCA2 (Supplementary Figure 1a), a domain thought to assist on the recognition of DNA secondary structures[3]. The same query sequence was used in a web-based prediction

tool for DNA binding residues, BindN[4], which predicted two clusters in this region highly enriched in residues with a strong propensity for DNA binding. A multiple sequence alignment using Clustal Omega[5] revealed that the DNA binding region is well conserved in mammals (Supplementary Figure 1b).

Biotinylated DNA pull-down assay

dT_{80} oligonucleotide with a 3'-biotin modification was purchased from Eurofins MWG and the dT_{40} was the same one used for EMSA. To perform the pull-down assay, 50 µl of magnetic streptavidin beads (MyOne Dynabeads, Thermo-Scientific) were resuspended and equilibrated in binding buffer (25 mM Tris Acetate pH 7.5, 1 mM $MgCl_2$, 2 mM $CaCl_2$, 1 mM DTT). Next, the dT_{80} oligonucleotide was incubated with the equilibrated beads for 2h at room temperature. Beads were then washed with binding buffer supplemented with 1 M NaCl, 0.3% NP40, and resuspended in binding buffer. Proteins were incubated with the beads-DNA (4.2 µM nt) at 37°C for 30 min and then washed with binding buffer supplemented with 1M NaCl and 0.3 % NP40 to avoid non-specific binding. The beads and the supernatant samples were then resuspended in SDS loading buffer, boiled at 95 °C and loaded on a 7.5% stain-free SDS gel (BioRad). For the competition assay with free ssDNA, dT_{40} was added to the protein sample before incubating with the beads-DNA. The reaction was followed as described above.

Supplementary References

1. Carreira, A. & Kowalczykowski, S. C. Two classes of BRC repeats in BRCA2 promote RAD51 nucleoprotein filament function by distinct mechanisms. *Proc Natl Acad Sci USA* **108,** 10448–10453 (2011).
2. Zhao, W. *et al.* Promotion of BRCA2-Dependent Homologous Recombination by DSS1 via RPA Targeting and DNA Mimicry. *Molecular Cell* **59,** 176–187 (2015).
3. Petrucco, S. Sensing DNA damage by PARP-like fingers. *Nucleic Acids Research* **31,** 6689–6699 (2003).
4. Wang, L. & Brown, S. J. BindN: a web-based tool for efficient prediction of DNA

and RNA binding sites in amino acid sequences. *Nucleic Acids Research* **34,** W243–8 (2006).

5. Sievers, F. *et al.* Fast, scalable generation of high-quality protein multiple sequence alignments using Clustal Omega. *Mol. Syst. Biol.* **7,** 539–539 (2011).
